# Decreased immunoglobulin G in brain regions of elder female APOE4-TR mice accompany with Aβ accumulation

**DOI:** 10.1186/s12979-018-0142-7

**Published:** 2019-01-25

**Authors:** Lihang Zhang, Juan Xu, Jinchao Gao, Peiqing Chen, Ming Yin, Wenjuan Zhao

**Affiliations:** School of Pharmacy, Shanghai Jiao Tong University, 800 Dongchuan Road, Shanghai, 200240 China

**Keywords:** Immunoglobulin G, Apolipoprotein E4, Ageing, Alzheimer’s disease

## Abstract

**Background:**

Apolipoprotein E4 (APOE4) and ageing are the most important known risk factors for late-onset Alzheimer’s disease (AD). In the present study, we determined the alterations of IgG, CD19, and Aβ in various brain regions of uninfected male and female APOE3- and APOE4-TR mice at the age of 3 and 10 months to elucidate impacts of AD risk factors on alterations of brain IgG.

**Results:**

Positive staining for IgG was distributed across the brain, including neocortex, entorhinal cortex, hippocampus, thalamus and cerebellum. IgG positive staining was mainly located on microglia, but not astrocytes. Some IgG positive neurons were also observed, but only in mediodorsal thalamic nucleus. Compared with APOE3-TR mice, 10-month-old female APOE4-TR mice had lower IgG level in AD susceptible brain regions such as neocortex, entorhinal cortex and hippocampus, but no significant changes in thalamus and cerebellum, two regions nearly intact in AD. In addition, the expression of CD19, a specific marker for mature B cells, was significantly reduced in the hippocampus of 10-month-old female APOE4-TR mice. Although there were no obvious differences in plasma IgG levels between APOE4- and age matched female APOE3-TR mice, significant decreased B cell amount in blood of 10-month-old female APOE4-TR mice have also been found. Moreover, more obvious positive staining for Aβ was observed in the cortex of 10-month-old female APOE4-TR mice than other groups.

**Conclusions:**

Our study demonstrated that AD risk factors were associated with IgG alterations in various brain regions, which might result from the defects of humoral immunity and lead to the impairment of IgG-mediated clearance of Aβ by microglia, therefore facilitated AD progression.

## Background

Alzheimer’s disease (AD) is the major age-related neurodegenerative disease, which is characterized by the accumulation of β-amyloid (Aβ) plaques and tau-laden neurofibrillary tangles [1–3]. It is still unsettled what causes Alzheimer’s disease. But certain factors like apolipoprotein E4 (APOE4) and ageing increase the risk of developing this incurable disease. Human APOE *ε4* is the first risk gene identified and remains the strongest genetic risk factor for AD, as it dramatically increases AD progression and decreases age of onset [4]. APOE4 coupled with aging significantly increases AD risk. Studies also showed that APOE4 confers greater risk of AD for women than men, with worse cognition deficits and neuropathology of plaques and tangles [5].

APOE *ε4* is one of three common alleles of the human APOE genes, the others are *ε2* and *ε3*. Unlike *ε4, ε3* allele is the most prevalent in the population, and *ε2* allele may play a protective role in AD progression. APOE isoforms differentially modulate β-amyloid precursor protein (APP) processing and Aβ synthesis (E4 > E3 > E2) [6]. Especially, they differentially affect the clearance of brain Aβ into glia or into the periphery, and APOE4 has much lower efficiency in Aβ clearance in mouse models that express human APOE gene [7, 8]. Studies from both AD patients and transgenic mouse models have demonstrated that APOE4 promotes Aβ accumulation and Aβ plaque loads in the brain. [9]. Microglial phagocytosis has been proposed as an Aβ-lowering mechanism of Aβ immunization in AD [10, 11]. Several lines of evidence suggest that peripheral IgG can reach the brain and bind to Fc receptors on microglia, promoting the clearance and phagocytosis of Aβ [12–14]. Intravenous or directly intracerebral immunoglobulin enhances the clearance of Aβ from the brain of AD patients or mouse models by mediating microglial phagocytosis of Aβ [12, 13, 15–18]. Although the primary outcomes of the Phase III pivotal study did not provide evidence that intravenous immunoglobulin (IVIg) was efficacious for treating symptomatic AD (cognitive decline) at the doses tested, IVIg treatment did result in clearing amyloid from the brain, and be efficacious for treating some subgroups of AD patients, particularly among APOE4 carriers, indicating that APOE4 carriers have specific humoral immunity affecting the IgG mediated clearance of Aβ by microglial phagocytosis in the brain [13, 19, 20]. In our previous study, we have found out that APOE4-TR mice had decreased levels of IgG and IgA in the brain compared with APOE3-TR control mice [21]. Moreover, Aβ clearance by microglia in APOE4-TR mice appeared to recede with more Aβ-positive neuron and less Aβ-positive microglia in the cortex after lentiviral Aβ_1–42_ injection [22, 23]. But Whether the APOE gene and Aging could affect the IgG levels in specific brain region then inducing the Aβ accumulation have not been reported yet.

The aim of our study was to determine whether APOE4, ageing will cause an alteration of IgG level and distribution in specific brain regions of the transgenic mice then where are the differences come from. Based on the result of the IgG levels changing, we finally observed the Aβ accumulation at certain area of the mice brain. These studies gave rise to our hypothesis that the alteration of IgG in the brain of APOE4 carriers might be a potentially critical way to promote Aβ accumulation and AD progression.

## Materials and methods

### Animals

APOE-TR mice, in which the endogenous mouse APOE gene was replaced by either human APOE3 or APOE4, were created by gene targeting [24]. PCR-RFLP analysis was used to determine the genotype of mice as described previously [25]. All animals were housed at the Shanghai Jiao Tong University Animal Care Center, under controlled temperature (22 ± 2 °C), with a 12-h light–dark cycle period and access to pelleted food and water ad libitum.

All the experiments were performed on gender balanced mice at the age of 3 or 10 months, and approved by the Shanghai Jiao Tong University Animal Care Committee. Every effort was made to reduce animal stress and to minimize animal usage.

### Flow cytometry of B-cell in the peripheral blood and speen

After mouse was anesthetized with ketamine (130 mg/kg, i.p.), 50 μl whole blood was taken from the orbital venous sinus and collected in a flow tube with anticoagulant. The blood cells were incubated with 500 μl (1:500 diluted in flow staining buffer) rat anti-mouse CD19-FITC (BioLegend) for 30 min at room temperature (RT) in the dark, and then 2 ml 1× RBC Lysis Buffer was added for 8 min at RT in the dark. Next, 2 ml flow staining buffer was added and centrifuged at 2000 rpm for 5 min. The supernatant was discarded. The pellet was washed two times by staining buffer, resuspended in 300 μl flow staining buffer, and measured by BD LSR Fortessa.

**Fig. 1:**
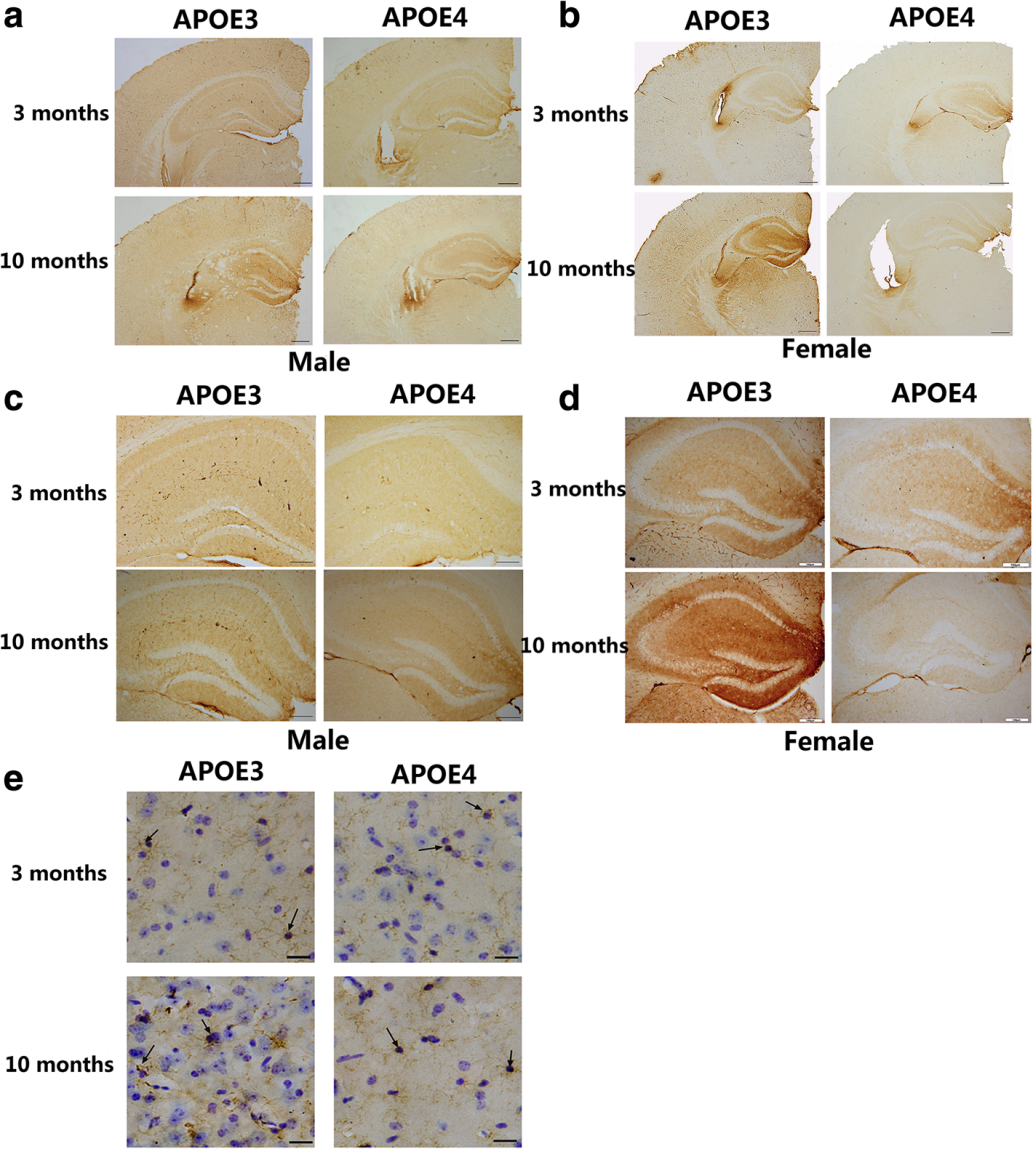
Distribution of IgG in the brain of male and female APOE-TR mice at the age of 3 and 10 months. **a** Representative images of IgG immunostaining on the brain sections from male APOE3- and APOE4-TR mice at the age of 3 or 10 months. Scale bar: 200 μm. **b** Representative images of IgG immunostaining on the brain sections from female APOE3- and APOE4-TR mice at the age of 3 or 10 months. Scale bar: 200 μm. **c**, **d** Representative images of IgG immunostaining in the hippocampus of male and female APOE-TR mice at the age of 3 or 10 months. Scale bar: 100 μm. **e** Combination of DAB-staining for IgG (brown) with Nissl staining (blue) in the brain of female APOE-TR mice. Arrows indicated that IgG-positive staining on glia-like cells. Scale bar: 20 μm

Single-cell suspensions from lungs and spleen were depleted of RBCs using hypotonic lysis. Cells were resuspended in PBS with 0.2% BSA and 0.1% sodium azide (FACS buffer). Single-cell suspensions were incubated for 15 min with anti-CD16/32 (Fc block) and stained for 30 min at 4 °C. DAPI, anti-mouse CD3 (17A2), CD19 (1D3), CD11b (M1/70), CD5 (53–7.3), andIgM (II/41) were purchased from eBioscience. Cells were analyzed on an LSR II (Becton Dickinson). More than 0.5310 5 cells were analyzed per sample, with dead cells excluded by DAPI + staining. Surface markeranalysis was performed using FlowJo software (TreeStar). B cells (CD19 +), B-1 B cells (CD19 +, sIgM +, CD11b +), B-1a B cells (CD19 +, sIgM +, CD11b+, CD5 +), and B-1b B cells (CD19 +, sIgM+, CD11b +,CD5 2) were identified with the appropriate gating. Gating strategy and controls are shown in Fig. 1.

Single-cell suspensions from spleen were depleted of RBCs using hypotonic lysis. Cells were resuspended in PBS with 0.2% BSA and 0.1% sodium azide (FACS buffer). Single-cell suspensions were incubated for 15 min with anti-CD16/32 (Fc block) and stained for 30 min at 4 °C with DAPI (eBioscience) or anti-mouse CD19.10000 cells were analyzed per sample, with dead cells excluded by DAPI^+^ staining. The data was analyzed with FlowJo software 7.0 (FlowJo).

### Tissue preparation

Mice were anesthetized with ketamine (130 mg/kg, i.p.) before intracardial perfusion with 0.01 M PBS. Brains were quickly removed, and hemispheres were separated. The neocortex, entorhinal cortex, hippocampus, thalamus and cerebellum was separated from the right hemisphere and then immediately flash-frozen in − 80 °C for subsequent biochemical analysis. The left hemisphere was post-fixed in 4% (wt / vol) paraformaldehyde for 24 h at 4 °C. After fixation, the left hemispheres were transferred to two sequential 30% sucrose solutions at 4 °C for 24 h, then were transferred to 0.01 M PBS for storage until sectioning. Coronal sections of the brain were cut (30 μm thick) on a freezing sliding microtome (Lecia CM1950). Subsequently, sections were stored in cryoprotectant (30% ethylene glycol, 30% glycerin, in 0.01 M PBS) at-20 °C until to further experiment.

### ELISA assay

Mouse blood from the orbital venous sinus was collected into anticoagulant tubes under general anesthesia with ketamine (130 mg/kg, i.p.) on ice and centrifuged at 800 rpm for 20 min at 4 °C. Plasma was obtained from the supernatant and diluted 1:10,000 as directed by manufacturer (Elabscience, Wuhan, China) before being loaded onto the plate. Brain tissues were extracted rapidly and immediately homogenized on ice. Total mouse IgG levels in various brain regions were examined by ELISA according to manufacturer’s protocol (Elabscience, Wuhan, China). Briefly, Samples were normalized to the total protein content. 100 μl of test samples were loaded into the wells of ELISA plate, incubated for 1.5 h for the antigens in the sample to fix to the walls of the well, and then rinsed with washing buffer. Next, enzyme linked antibody was placed into the washed wells and allowed to stay for 0.5 h. The wells were rinsed again. ELISA substrate was loaded and incubated for 15 min. Place the plate into the socket of ELISA instrument and take the reading after detection.

### Immunohistochemistry—DAB staining

Immunostaining for IgG was carried out as previously described [26, 27]. Briefly, the 30 μm-thick sections were washed in 0.01 M PBS three times in order to wash out cryoprotectant and then incubated in 3% hydrogen peroxide (methanol: 30% H_2_O_2_ = 1:1 in 0.01 M PBS) for 20 min, permeabilized with PBS containing 0.25%Triton X-100 (PBST, 3 × 10 min). The sections were blocked in 5% normal goat serum at RT for 1 h, followed incubation with HRP-conjugated goat anti-mouse IgG (Boster Biological Technology, Wuhan, China) overnight at 4 °C. Free-floating sections were subsequently washed in PBS three times. Sections were incubated in 0.05% diaminobenzidine (DAB) to visualize reaction products and then washed with PBS (3 × 5 min), mounted to a glass slide. Then sample was dried in air overnight, dehydrated using graded ethanol, cleared in xylene before cover-slipped with Neutral balsam. Images were captured under a light microscope (Olympus Corporation, Tokyo, Japan).

Immunostaining for Aβ was followed with the above procedures, incubated with primary antibody MOAB2 (Novus) followed by HRP-conjugated goat anti-mouse IgG secondary antibody.

### Nissl staining

DAB stained sections were counter-stained with Nissl staining. Coverslips were lightly removed with xylene, rehydrated with decreasing grade ethanol. Sections were washed in 0.01 M PBS for 5 min, immersed in 0.1% cresyl violet for 5 min at RT, rinsed in distilled water for 3 min, then successively dehydrated with 70, 95, and 100% ethanol, treated with xylene and cover-slipped with permount.

### Immunofluorescence staining

Immunofluorescence staining of CD19 with specific cell-type antibodies was performed to determine the existence of CD19 expression in the brain. Brain sections was coincubated with CD19 (Abcam) antibody and NeuN (Abcam) antibody at 4 °C overnight. After that, sections were rinsed 3 times with 0.01 M PBS and incubated with anti-mouse IgG-Alexa Flour 488 (Thermo Scientific) and anti-rabbit IgG-Alexa Flour 594 at RT for 2 h in the dark. Then, incubated in the DAPI solution for 5 min in the dark, washed three times and mounted with anti-fluorescence quencher. Images were taken on a fluorescence microscope (Olympus Corporation, Tokyo, Japan).

Double immunofluorescence staining of IgG with specific cell-type antibodies was performed to determine the cellular localization of IgG in the brain. Brain sections was incubated with two antibodies: IgG (Thermo Scientific) and either Iba1 (Dako), NeuN (Abcam), or GFAP (Proteintech) and the following steps are the same as described above.

### Western blotting analysis

Frozen neocortex, entorhinal cortex, hippocampus, thalamus and cerebellum tissues were homogenized in eight times their volume of RIPA buffer on ice cold for 30 min to completely crack. Homogenates were centrifuged at 12, 000 rpm for 20 min at 4 °C. The supernatant was reserved for Western blotting. Total protein concentration was determined by BCA Protein Assay Kit (Beytoine) and adjusted to 5 mg/ml.

Samples were separated by SDS-PAGE followed by a transfer to polyvinylidene difluoride (PVDF) membranes (Millipore). The membranes were blocked with 5% BSA (Amerso) in TBST buffer at RT for 2 h, and incubated overnight at 4 °C with anti-CD19 (Abcam), β-actin (Bioworld) and GAPDH (Bioworld), then incubated 2 h with HRP-conjugated antibody at RT. The protein bands were visualized using the ECL chemiluminescent substrate (Pierce).

### Statistical analysis

Data was presented as mean ± S.E.M. Statistical analysis was performed using the GraphPad Prism 5.0 software. ANOVA followed by Bonferroni’s post hoc test was used to calculate statistical differences among groups. *P* values that are less than 0.05 were defined as significant difference.

## Results

### Distribution of IgG in the brain of APOE-TR mice

Regional distribution of IgG in the brain of APOE-TR mice was obtained with a HRP-conjugated goat anti-mouse IgG antibody. Significant IgG positive staining was widespread throughout the brain including neocortex, entorhinal cortex, hippocampus, thalamus and cerebellum in adjacent cryosections. One particularly strong IgG-positive staining was found out around ventricles, a pathway through which peripheral IgG can enter brain (Fig. 1a, b). Compared with the younger mice, 10-month-old APOE3-TR mice showed higher IgG immunostaining intensity in the whole brain (Fig. 1a, b), especially in hippocampus (Fig. 1c, d). Male APOE4-TR mice had similar features of IgG staining as APOE3-TR mice (Fig. 1a, b). However, 10-month-old female APOE4-TR mice, unlike 10-month-old female APOE3-TR mice, did not show an increased IgG staining, but an obviously weaker staining in the brain (Fig. 1a, b), especially in hippocampus (Fig. 1a-d), a brain region critical for learning and memory and vulnerable to be affected at early stages of AD [28].

Furthermore, combination of DAB-labeling immunohistochemistry for IgG with Nissl staining exhibited that IgG immunoreactivity was not only scattered extracellularly but also localized on glia-like cells, including both cell bodies and projects as arrows indicated in Fig. 1e. Additionally, positive IgG staining in blood vessels was also observed (not shown).

### Alteration of IgG levels in various brain regions associated with ageing, sex and APOE genotype

**Fig. 2:**
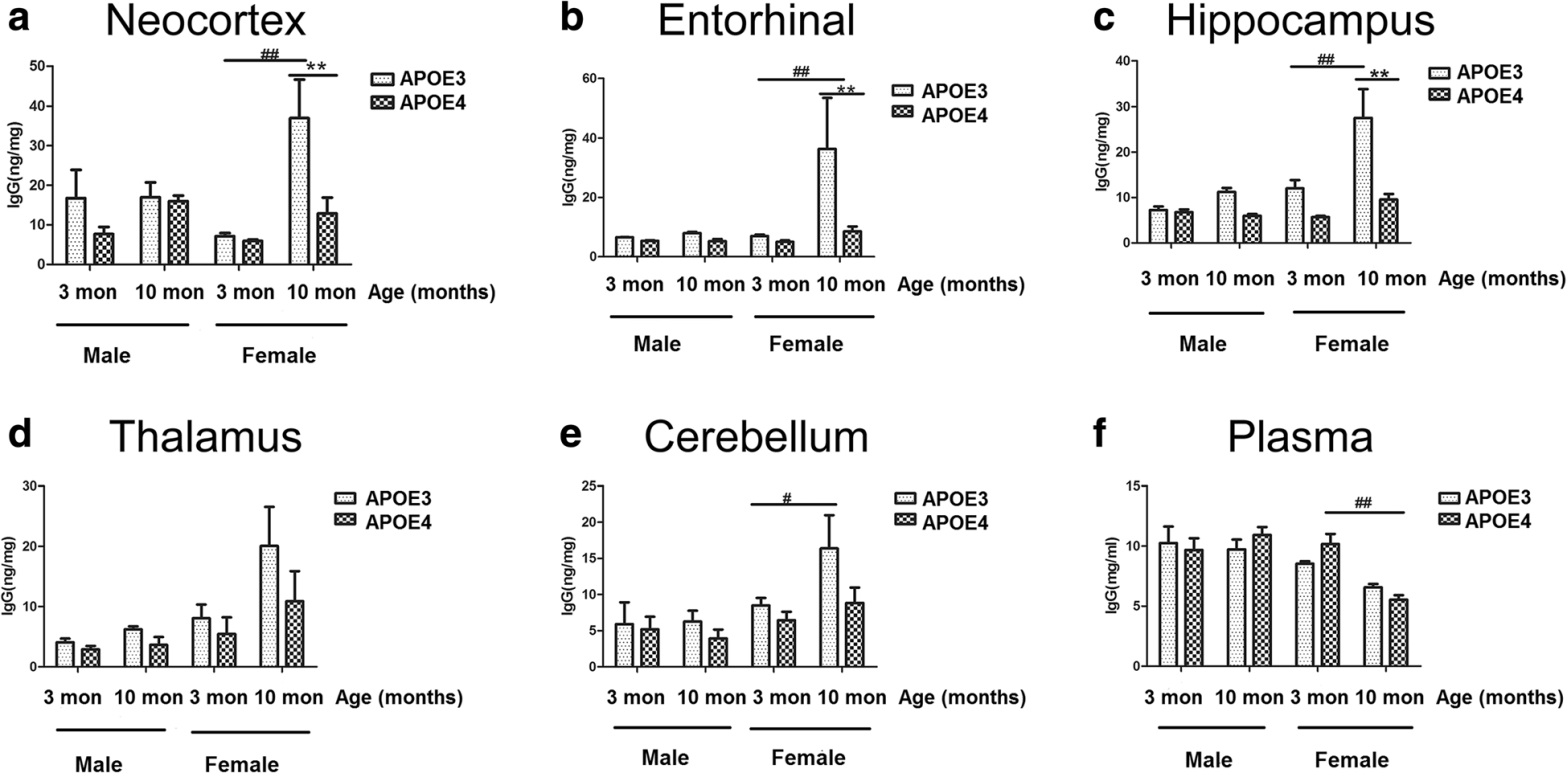
IgG levels in the brain regions of APOE3- and APOE4-TR mice. The total IgG levels in neocortex (**a**), entorhinal cortex (**b**), hippocampus (**c**), thalamus (**d**), cerebellum (**e**) and plasma (**f**) of 3-, 10-month-old male and female APOE3- and APOE4-TR mice. Data were expressed as mean ± SEM and analyzed by ANOVA followed by Bonferroni post-test, Asterisks (*) and pound signs (#) indicate significant difference to APOE3-TR and 3-month-old control mice as indicated, respectively. **P < 0.01, ##*P* < 0.01, #*P* < 0.05, *n* = 4–6

In order to determine the IgG levels in the brain regions of APOE-TR mice, tissues from neocortex, entorhinal cortex, hippocampus, thalamus and cerebellum were isolated and the total IgG levels were measured by ELISA. As shown in Fig. 2, there were no significant differences among the IgG levels in various brain regions when compared by age (3-month-old vs. 10-month-old) or APOE genotype (APOE3 vs. APOE4) (Fig. 2a-e). Consistent with the observation of IgG immunostaining and our previous studies [21], the IgG levels in neocortex, entorhinal cortex, hippocampus and cerebellum of 10-month-old female APOE3-TR mice were significantly higher than 3-month-old female APOE3-TR mice, while the IgG levels in various brain regions of 10-month-old female APOE4-TR mice did not increase as the female APOE3-TR mice at the same age. 10-month-old female APOE4-TR mice had significantly lower IgG levels in neocortex, entorhinal cortex and hippocampus, areas of the brain commonly affected by AD, than those of APOE3-TR control mice (Fig. 2a-e). But there were no obvious differences in IgG levels of thalamus or cerebellum, which are nearly intact in early AD, between10-month-old female APOE4-TR mice and APOE3-TR control mice (Fig. 2a-e).

In addition, the levels of IgG in plasma were surveyed as well. As shown in Fig. 2f, there were no significant differences among the IgG levels in plasma from male mice compared by age or APOE genotype. 10-month-old female APOE3-TR mice did not show increased IgG level in plasma, unlike those in brain regions (Fig. 2f). IgG level in plasma of 10-month-old female APOE4-TR mice were significantly lower than that of 3- month- old control mice (Fig. 2f**)**.

### In the brain, IgG was present mainly on microglia, some neurons in mediodorsal thalamic nucleus, but not astrocytes

**Fig. 3:**
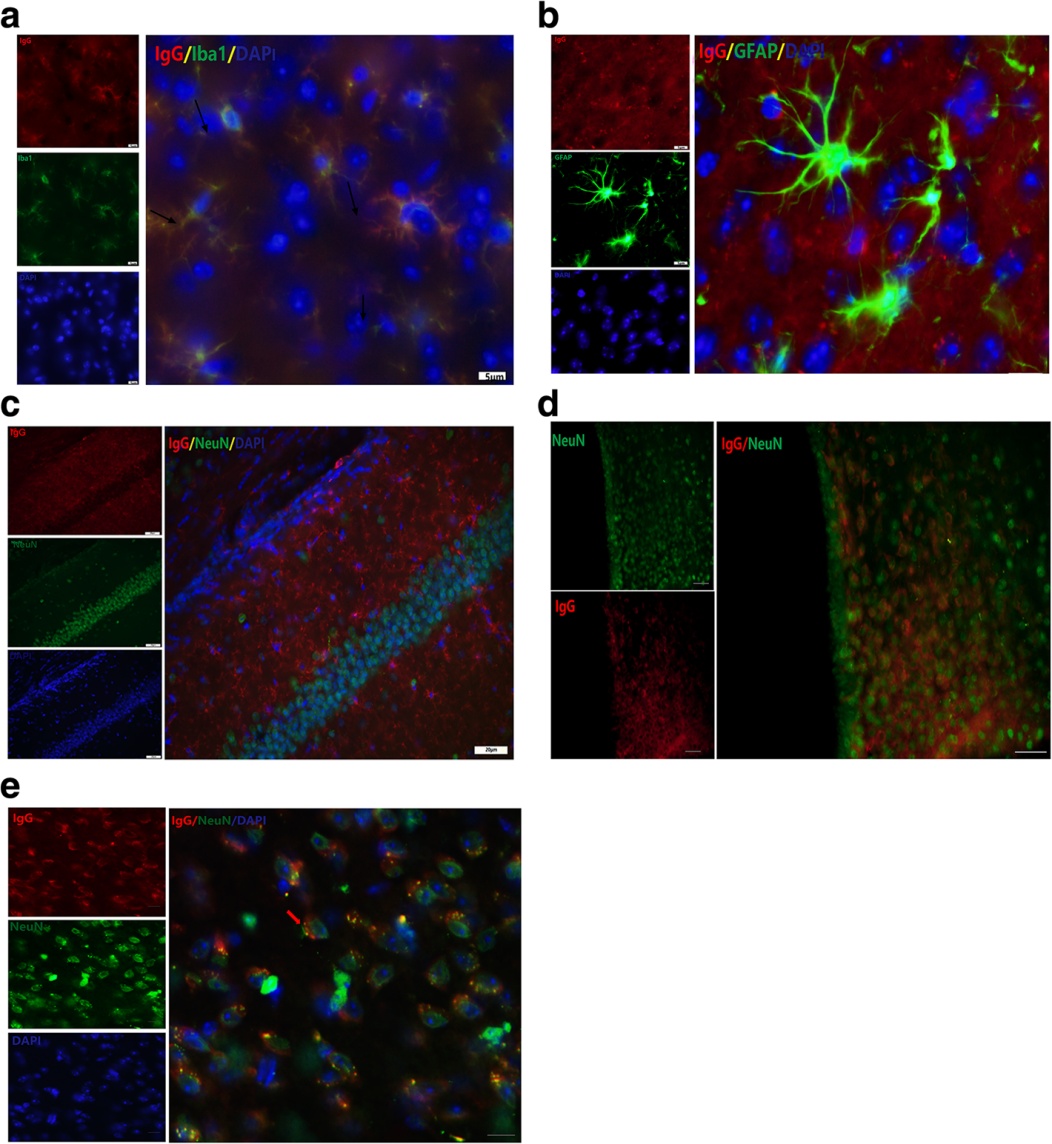
The presence of IgG on specific cell-type in the brain. **a** Representative images of double-immunofluorescent staining for IgG (red) and microglia (Iba1, green). Arrows pointed to the IgG positive immunoreactivity on microglia. Scale bar: 5 μm. **b** Representative images of double-immunofluorescent staining for IgG (red) and astrocyte (GFAP, green). Scale bar: 5 μm. **c** Representative images of double-immunofluorescent staining for IgG (red) and neuron (NeuN, green) in hippocampus. Scale bar: 20 μm. **d**, **e** Representative images of double-immunofluorescent staining for IgG (red) and neuron (NeuN, green) in mediodorsal thalamic nucleus. Scale bar: 20 μm (**d**), 5 μm (**e**)

DAB immunohistochemistry for IgG with Nissl staining indicated that IgG immunoreactivity has some morphological similarity with glial cells. To determine the specific cell type that IgG was located in the brain, we performed double-immunofluorescence with IgG antibody and specific antibodies to identify different neuronal types. As shown in Fig. 3a, IgG (red) in brain was mainly presented on microglia (Iba1, green), located in perinuclear compartments and projections. No IgG positive staining was observed on astrocytes (GFAP, green) in the mouse brain examined (Fig. 3b). IgG immunoreactivity was not found on neurons (NeuN, green) in most brain regions including cortex and hippocampus (Fig. 3c), except mediodorsal thalamic nucleus (Fig. 3d, e), which was concerned with visual recognition-related memory and spatial memory [29]. In mediodorsal thalamic nucleus, IgG positive immunostaining was mainly located perinuclearly in neurons (Fig. 3e).

### B lymphocyte amounts in circulating blood, spleen and the expression of CD19 in various brain regions associated with ageing, sex and APOE genotype

**Fig. 4:**
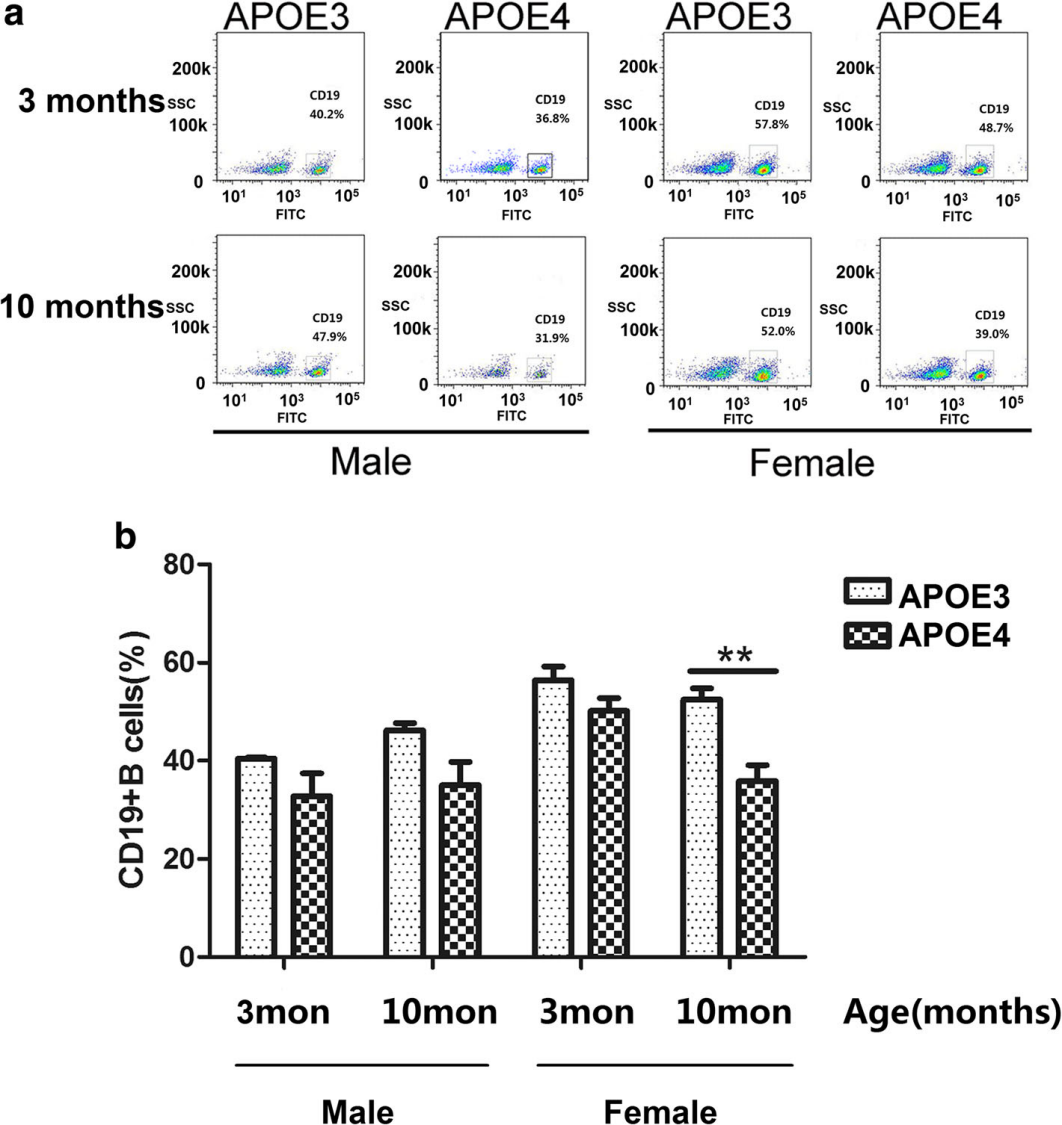
B lymphocyte amounts in the blood of 3-, 10-month-old male and female APOE3- and APOE4-TR mice. **a** Representative CD19+ B cell analysis diagram by flow cytometry in blood of different groups. **b** The percentages of CD19 + B cell in blood of different groups. Data were expressed as mean ± SEM and were analyzed by ANOVA followed by Bonferroni post-test, Asterisks (*) and pound signs (#) indicate significant difference to 10-month-old female APOE3-TR and 3-month-old female APOE4-TR control mice as indicated, respectively. **P < 0.01, #*P* < 0.05, *n* = 4–6

**Fig. 5:**
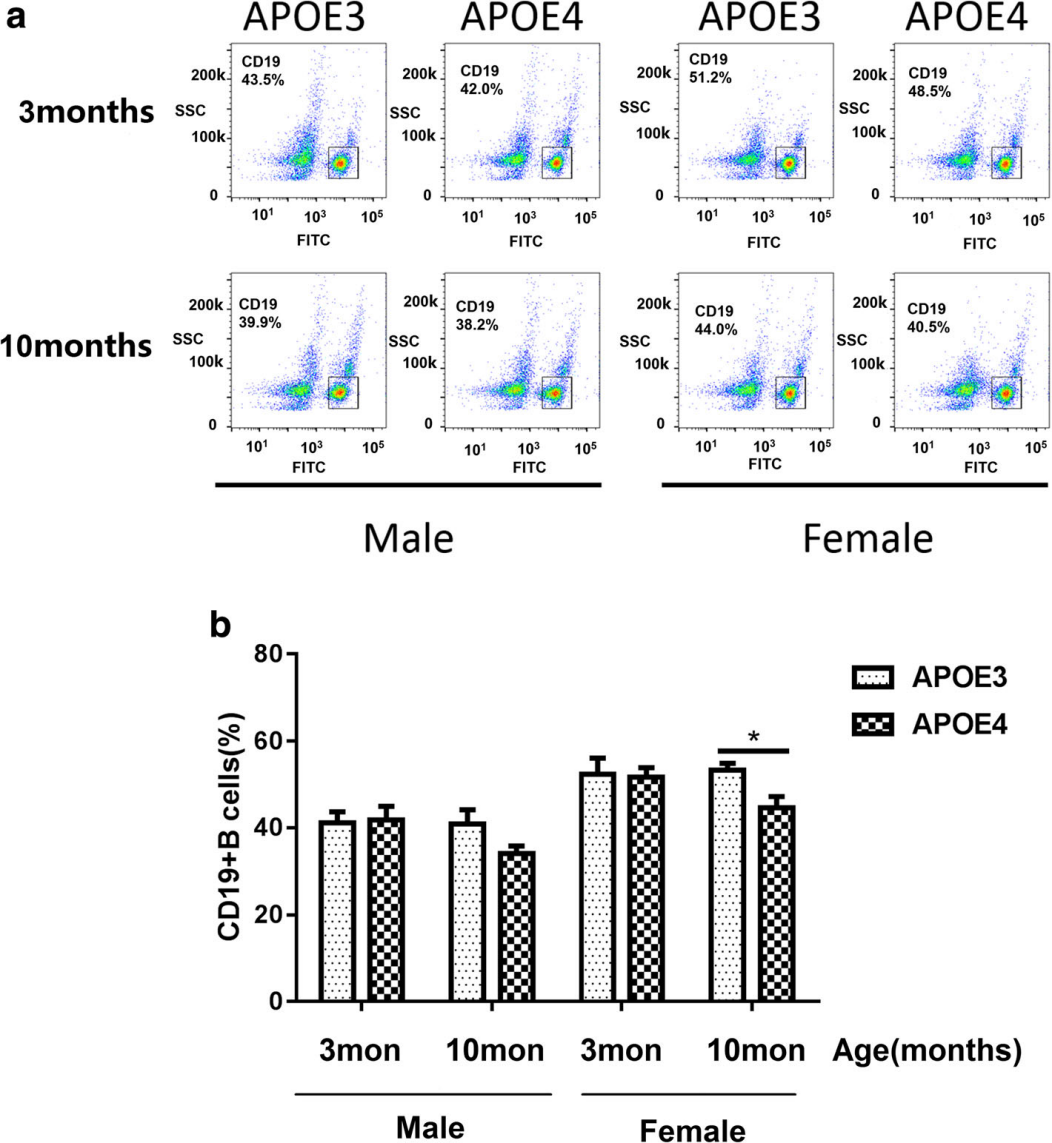
B lymphocyte amounts in the spleen of 3-, 10-month-old male and female APOE3- and APOE4-TR mice. **a** Representative splenic CD19+ B cell analysis diagram by flow cytometry of different groups. **b** The percentages of splenic CD19 + B cell of different groups. Data were expressed as mean ± SEM and were analyzed by ANOVA followed by Bonferroni post-test, Asterisks (*) indicate significant difference to 10-month-old female APOE4-TR mice, respectively. *P < 0.05, *n* = 4–6

As B cells play a critical role in antibodies production and humoral immunity, we also measured B cell percentage in the peripheral blood by flow cytometry. B cells were labeled with CD19, a specific B cell biomarker found on mature and activated B cells but not plasma cells. The percentages of CD19+ B cells in blood appeared lower in APOE4-TR mice than APOE3-TR mice of the same sex and age, especially in 10-month-old female groups (Fig. 4). The percentage of CD19+ B cell in blood of 10-month-old female APOE4-TR mice was significantly lower than that of 10-month-old female APOE3-TR or 3-month-old female APOE4-TR control mice (Fig. 4b). And the percentage of CD19+ B cells in spleen was lower in 10 month old female APOE-4 TR mice, which was similar to the results from the blood (Fig. 5a, b).

**Fig. 6:**
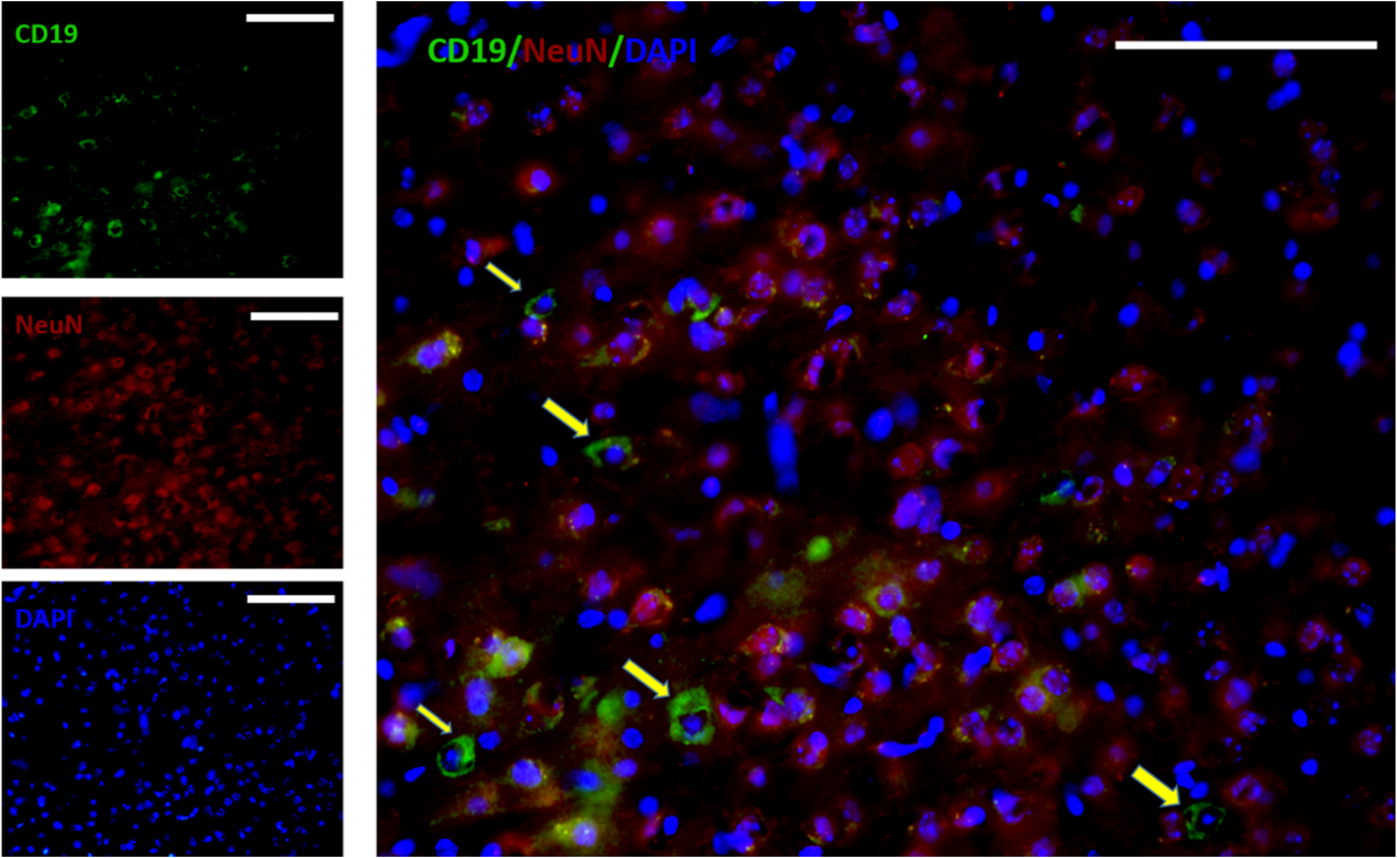
Expression of CD19 on the cell of mouse brain. Double-immunofluorescent staining for CD19 (green), neuron (NeuN, red) and nucleus (DAPI, blue). Arrows pointed to the CD19 positive immunoreactivity on specific cells. Scale bar: 100 μm

**Fig. 7:**
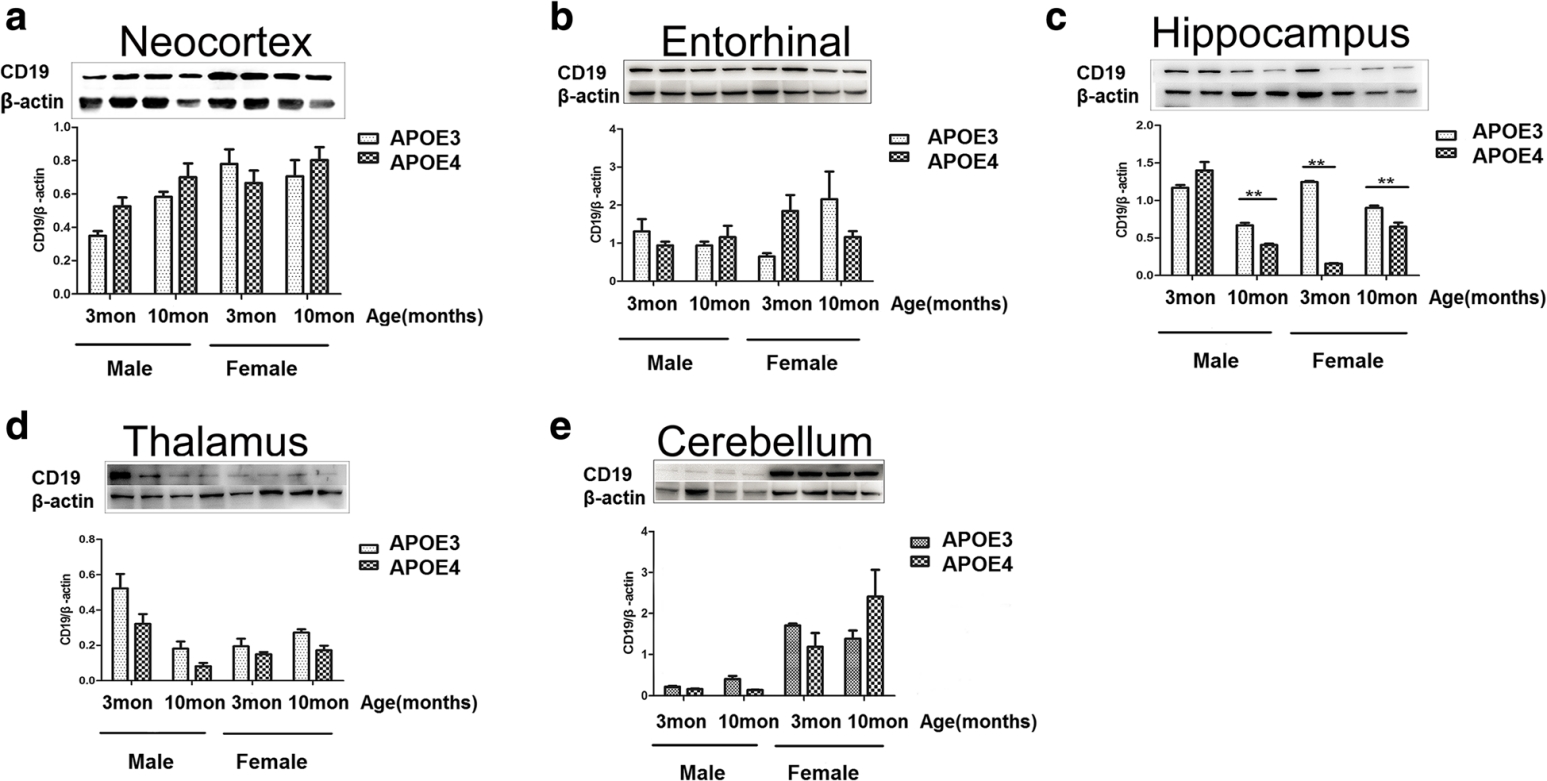
Expressions of CD19 in brain regions of 3-, 10-month-old male and female APOE3- and APOE4-TR mice by western blotting. **a-e** Representative images (top) and quantification (bottom) of western blotting for CD19 (95 kDa) in neocortex (**a**), entorhinal cortex (**b**), hippocampus (**c**), thalamus (**d**) and cerebellum (**e**). Data were expressed as mean ± SEM and were analyzed by ANOVA followed by Bonferroni post-test, Asterisks (*) indicate significant difference to female APOE3-TR control mice as indicated. **P < 0.01, *n* = 4–6

Expressions of CD19 on specific cells but not neurons (probably filtrated B cell) in the brain (Fig. 6). And the expressions of CD19 in various brain regions were also determined by western blotting. As shown in Fig. 7a, b, the expressions of CD19 in neocortex and entorhinal cortex did not show significant differences among groups when compared by age (3-month-old vs. 10-month-old), sex (male vs. female) or APOE genotype (APOE3 vs. APOE4). In hippocampus, the expression of CD19 was significantly lower in 3-, 10-month-old female and 10-month-old male APOE4-TR mice than in compared APOE3-TR mice as indicated in Fig. 7c. The expression of CD19 in thalamus appeared similar pattern as in hippocampus with lower, but not significantly, level in APOE4-TR mice than in compared APOE3-TR mice as indicated in Fig. 7d. Immunofluorescent staining for CD19 revealed that most CD19 positive cells distributed around ventricles (data not shown), the expressions of CD19 in hippocampus and thalamus may come from functional lymphatic vessels lining the dural sinuses nearby cerebrospinal fluid. But, on the other hand, the expressions of CD19 in cerebellum showed a different pattern with higher levels in female mice. Especially, 10-month old female APOE4-TR mice had the highest CD19 expression in cerebellum, although there were no significant differences compared to 10-month old female APOE3-TR or 3-month old female APOE4-TR mice (Fig. 7e). Thus, there may be different sources/pathways of CD19 positive cells and CD19 production in cerebellum.

### Aβ accumulation in the brains of 3-, 10-month-old male and female APOE3- and APOE4-TR mice

**Fig. 8:**
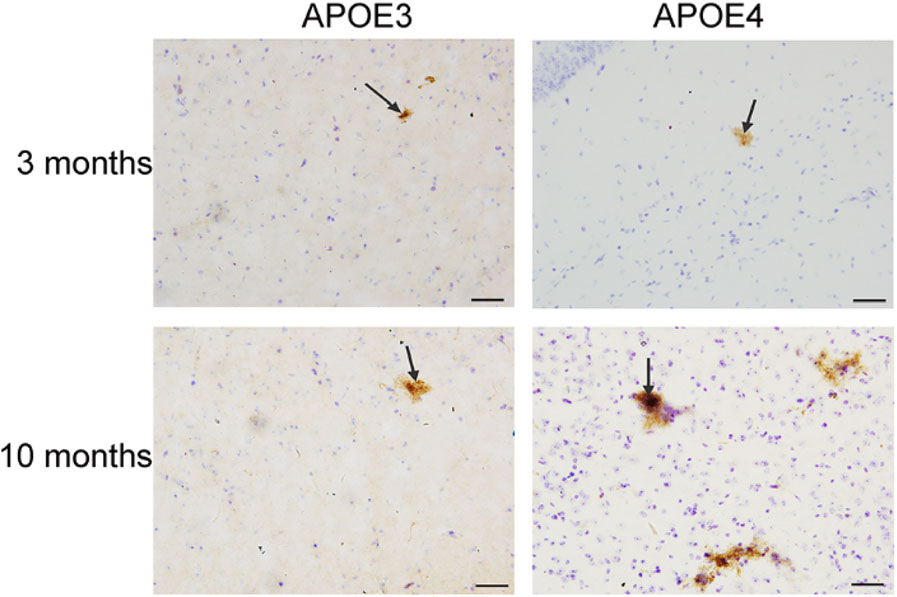
Aβ accumulation in the cortex of uninfected, non-AD female APOE3- and APOE4-TR mice at the age of 3 or 10 months. Representative images of DAB-staining for Aβ_42_ (MOAB2, brown) with Nissl staining (blue) showed the Aβ accumulation (arrowhead) in the cortex of each group. Scale bar: 20 μm

Aβ accumulation in the brain was measured by DAB immunostaining with MOAB2, a pan-specific antibody to Aβ_42_. There was pretty rare MOAB2 positive staining in the whole brain of the mice detected (uninfected, non-AD male and female APOE3- and APOE4-TR mice at the age of 3 and 10 months). Only a few sporadic Aβ accumulations mainly in the cortex were observed, and they were more obvious and easier to be found in the cortex of 10-month-old female APOE4-TR mice (Fig. 8).

## Discussion

In the present study, we found that IgG distributed throughout the brain of uninfected, non-AD male and female APOE3- and APOE4-TR mice at the age of 3 and 10 months, located mainly on microglia as well as some neurons in mediodorsal thalamic nucleus. The alteration of IgG levels in various brain regions was associated with age, sex and APOE genotype. 10-month-old APOE3-TR mice had highest IgG levels in various brain regions, while the IgG levels were significantly lower in brain regions severely affected by AD (neocortex, entorhinal cortex and hippocampus), but not in relatively intact brain regions (thalamus and cerebellum) of 10-month-old female APOE4-TR mice. Flow cytometry showed less B cell amounts in blood of 10-month-old female APOE4-TR mice than those in female APOE3-TR mice at the same age and spleen B cell show similar decrease in 10 month old female APOE4-TR mice than female APOE3-TR mice which indicate the reduction is a systemic loss. The brain expression of CD19, a specific marker expressed on mature and activated B cells, was also significantly reduced in hippocampus of 10-month-old female APOE4-TR mice. In addition, there are more obvious Aβ accumulations in the cortex of 10-month-old female APOE4-TR mice than those in other groups. Overall, the present study for the first time, demonstrated that AD risk factors such as APOE4, aging and female, were related to IgG alterations in certain brain regions, potentially due to abnormalities of B cells and humoral immunity, and result in impairment of IgG-mediated Aβ clearance by microglia, which may finally lead to AD.

As described by Taconic Biosciences, APOE-TR (APOE3- and APOE4-TR) mice were created by gene targeting and carry APOE3 or APOE4 in place of the endogenous murine apoE gene. The mice express the human apoE protein at physiological levels and retain the endogenous regulatory sequences required for modulating apoE expression. Therefore the mice have identical levels of apoE expression in all normally apoE-occurring mouse cell types. This provides a complete in vivo system that allows for direct comparison studies to measure any apoE isoform-specific effects.

ApoE is a plasma protein involved in cholesterol transport, with three human isoforms (E2, E3, and E4). Differences in serum lipid transport compared to wild-type mice, APOE2-TR mice exhibit markedly abnormal plasma lipid profiles and provide a model for human type III hyperlipoproteinemia with premature atherosclerosis. Whereas the APOE3- and APOE4- TR mice have normal plasma cholesterol and triglyceride levels on a normal diet, although they are susceptible to diet-induced atherosclerosis.

Among the three human isoforms, E3 is the most common isoform, expressed by almost 80% of the human population. E4 occurs in approximately 14% of the human population, which is the strongest genetic risk factor for late-onset AD. APOE3- and APOE4-TR mice are mostly used in exploring the roles for direct comparison studies to measure the effects human apoE3 and apoE4 in normal brain physiology and Alzheimer’s disease, while the effects of murine apoE of wild type mice were not generally covered. In our study, plasma ApoE levels in 3-month male, 3-month female 10-month male and 10-month female APOE3-TR mice are 10.25 ± 1.90, 8.53 ± 0.19, 9.73 ± 1.76 and 6.57 ± 0.21 mg/ml, respectively, which are almost in the typical range (about 7 to 10 mg/ml.) of mouse plasma IgG. In addition, the IgG levels in brain regions of APOE3-TR mice we obtained in this experiment are in a range of 5–20 ng/mg protein, at a similar level to those in brain regions of C57BL/6 mice [30], from which APOE-TR mice originate. From these results, we could speculate that the IgG level in APOE3-TR mice is similar to that in wildtype mice.

Recently, the presence of IgG in brain, including healthy brains, was observed in several studies [31, 32]. Similarly, we demonstrated that IgG distributed throughout the brain of uninfected, non-AD male and female APOE-TR mice and IgG levels in various brain regions of 10-month-old female APOE3-TR mice were higher than those of other groups. The presence of IgG in brain was often attributed to blood brain barrier breakdown in neurovascular injury [33, 34]. Studies revealed increased hippocampal IgG expression in senescent female rats was paralleled by disruption in claudin-5 distribution, a microvessel tight junction protein associated with ovarian aging, when compared young and senescent female with those of age-matched male rats. The similar findings were achieved from parallel studies in post-menopausal women compared to pre-menopausal women [35]. In addition, estrogen induced polyclonal B cell activation with increased expression of antibodies in normal mice [36]. Females had higher IgG levels and mounted stronger immune responses following immunization or infection than males [37, 38]. Thus, increased IgG levels in the brain of 10-month-old female APOE3-TR mice were presumably due to the age- and estrogen- dependent dysfunction of the endothelial barrier, which may facilitate the extravasation of plasma IgG into the brain.

However, 10-month-old female APOE4-TR mice did not show increased IgG levels in brain regions as the female APOE3-TR mice at the same age. This finding was consistent with our earlier study that APOE4-TR mice had lower IgG levels in the brain than APOE3-TR mice [21]. In this study, we further determined that the alterations of IgG in the brain of 10-month-old female APOE4-TR mice were region-specific, with significant lower IgG levels in AD vulnerable areas like neocortex, entorhinal cortex and hippocampus, but not in AD relatively intact brain regions like thalamus and cerebellum, suggesting APOE4, together with ageing and sex, affecting IgG levels in certain brain regions. In fact, the impact of APOE on brain IgG was once involved in Fernandez ‘s study, in which they reported increased brain levels of IgG and upregulation of activating Fc receptors in the brain of APOE knockout (APOE−/−) mouse, a well-established model of hypercholesterolaemia [38]. They considered hypercholesterolaemia was associated with increased levels of IgG against oxidized lipoproteins. This is a reasonable point, but we are not convinced by it. Although the origin and function of brain IgG is not clear now, it is thought that brain IgG generally comes from following sources: (1) plasma IgG which may enter the brain by crossing the BBB [33, 34]; (2) be synthesized in neurons [39]; (3) be produced by B cells that have transmigrated from the blood into the brain perivascular space [40, 41]. As discussed above, 10-month-old female APOE3-TR mice has elevated brain IgG on account of damages in the blood brain barrier and increasing extravasation of plasma IgG. Besides, IgG is also indicated being synthesized in neurons and neuron-derived IgG is involved in active immunity, neural development and protecting neurons from inflammatory injury or cytotoxicity by neutralizing complement to maintain the stability of the nervous system [39, 42, 43]. Greater expression of IgG was found in the older male brain than young adult males, which was thought to be indicative of a compensatory, trophic response [33, 34]. IgG was also seen on neurons in the current study, but the IgG positive neurons were not widely distributed, only limited in mediodorsal thalamic nucleus, an area associated with visual recognition-related memory and spatial memory [29], indicating neuron-related IgG may be associated with AD. The actual role of neuron-related IgG in mediodorsal thalamic nucleus is unknown but worth further study. Therefore, the reduced IgG in AD vulnerable brain regions of 10-month-old female APOE4-TR mice suggested that APOE4 may impair the level of plasma IgG or the production of IgG in brain. Consistently, we found the IgG levels, as well as the amounts of CD19 + B cell, were decreased in blood of 10-month-old female APOE4-TR mice, indicating the reduced production of IgG in blood of these mice. Moreover, B cells was indicated entering the brain through cerebrospinal fluid, choroid plexus or deep cervical lymph nodes pathway and staying within normal brains as an activated phenotype in very low numbers [44, 45]. Brain antigens, such as Aβ, APOE/Aβ complex, may be recognized and presented by B cells in the brain which then were stimulated to proliferate into antibody-secreting plasma cells in cervical lymph nodes and deep cervical lymph nodes [46, 47]. In the present study, female APOE4-TR mice showed decreased expressions of CD19, a specific B cell biomarker found on mature and activated B cells but not plasma cells, in regions near ventricles, especially in AD hippocampus (an area susceptible to AD). This is likely due to the impaired antigen recognition or presentation in these mice and APOE4 may be involved in these processes. However, 10-month old female APOE4-TR mice did not show decreased expressions of CD19 in cerebellum which is relatively intact in AD, as in other brain regions, indicating APOE4 may differentially affect B cells in various brain regions of the mice which may influence their susceptibility to AD.

Additionally, we determined that most IgG in the brain was on microglia. Microglia was known to display enhanced phagocytic activities through binding to the Fc region of an antibody. IgG was a potent stimulus for microglia activation, which can promote microglia-mediated clearance of Aβ [48]. Studies demonstrated that Aβ was eliminated by microglia phagocytosis in physiological conditions or at the early stages of AD, but as the disease progresses, the phagocytosis of microglia decreased at the advanced stage of AD [49]. In the current study, we found more Aβ accumulations with less IgG levels in the brain of 10-month-old female APOE4-TR mice. Our findings are consistent with earlier studies that depletion of B cells and their appropriate activation by T cells led to the loss of adaptive-innate immunity, reduced capacity of microglial phagocyte and increased Aβ pathology in Rag-5xfAD mice, while treatment with IgG enhanced Aβ clearance in Rag-5xfAD mice or microglial cells [31]. Taken together, these data suggest IgG mediated Aβ clearance by microglia was impaired in the older female APOE4-TR mice, as brain IgG were mainly on microglia. Actually, a phase 3 trial of IVIg immunotherapy for AD showed promised results in APOE4 carriers with decreased Aβ_42_ levels in plasma and improved cognition [18]. But so far, neither the mechanisms of immunotherapy for AD nor the neuroimmunological changes in AD brains are clear. Our study provides a novel potential neuroimmunologic mechanism for AD pathogenesis and immunotherapy, especially for aged female APOE4 carriers.

## Conclusion

In our study, for the first time, we demonstrated that AD risk factors, APOE4, aging and female, might undermine IgG production and/or its entrance into the brain, which brought about changes of IgG distributions and levels in brain regions, especially in AD susceptible regions like hippocampus and cortex. These might damage IgG medicated Aβ clearance by microglia, then induce Aβ accumulation and facilitate AD progression. Our study also suggested a novel neuroimmunologic mechanism of AD pathogenesis, especially for aged female APOE4 carriers, which may be indicative for AD immunotherapy and drug development.

## Abbreviations

AD: Alzheimer’s disease
APOE: Apolipoprotein E
APP: β-amyloid precursor protein
Aβ: β-amyloid
BBB: Blood brain barrier
IgG: Immunoglobulin G
IVIg: Intravenous immunoglobulin
TR: Targeted replacement
